# NLRP3 Marks an Inflammatory Neutrophil–NETosis–Pyroptosis Network in Human Ischemic Stroke Thrombi

**DOI:** 10.3390/cells15171597

**Published:** 2026-09-02

**Authors:** Maximilian Bellut, Milena Strootmann, Fabian Essig, Guido Stoll, Marius L. Vogt, Konstanze Guggenberger, Mirko Pham, Alexander M. Kollikowski, Michael K. Schuhmann

**Affiliations:** 1 Department of Neurology, University Hospital Würzburg, 97080 Würzburg, Germany; strootmann_m@ukw.de (M.S.); essig_f@ukw.de (F.E.); schuhmann_m@ukw.de (M.K.S.); 2 Institute for Experimental Biomedicine, University Hospital Würzburg, 97080 Würzburg, Germany; stoll_g@ukw.de; 3 Department of Neuroradiology, University Hospital Würzburg, 97080 Würzburg, Germany; vogt_m2@ukw.de (M.L.V.); guggenberg_k@ukw.de (K.G.); pham_m@ukw.de (M.P.); kollikowsk_a@ukw.de (A.M.K.)

**Keywords:** ischemic stroke, large vessel occlusion, mechanical thrombectomy, NLRP3 inflammasome, immunothrombosis, neutrophil extracellular traps (NETs), pyroptosis, danger-associated molecular pattern (DAMP), Gasdermin D (GSDMD), cerebral thrombus

## Abstract

**Highlights:**

**What are the main findings?**

- Human ischemic stroke thrombi harbor an NLRP3-centered inflammatory network closely associated with neutrophils, NETosis, HMGB1, and GSDMD.
- ASC-positive specks are frequently present in thrombus-associated neutrophils, providing histological evidence consistent with local inflammasome assembly.

**What are the implications of the main findings?**

- Stroke thrombi represent biologically active immunothrombotic structures in which inflammasome-associated signaling converges with neutrophil-driven inflammation.
- Thrombus-associated NLRP3 reflects local inflammatory thrombus biology rather than clinical stroke severity or functional outcome.

**Abstract:**

Background: Human ischemic stroke thrombi are increasingly recognized as biologically active immunothrombotic structures. While the NLRP3 inflammasome contributes to experimental stroke pathology, its role within human cerebral thrombi remains poorly understood. Methods: Thrombi retrieved by mechanical thrombectomy from 34 patients with acute ischemic stroke caused by large vessel occlusion were analyzed by immunofluorescence for NLRP3, ASC, neutrophils (CD66B), NETosis (H3CIT), HMGB1, and Gasdermin D. Results: NLRP3 was detected in all thrombi and correlated with neutrophil abundance, NETosis, HMGB1, and GSDMD independently of fibrin content. A majority of CD66B-positive neutrophils exhibited ASC immunoreactivity. Neither NLRP3 nor the inflammatory thrombus signature was associated with stroke severity, infarct extent, or functional outcome. Conclusions: Human ischemic stroke thrombi contain an NLRP3-associated neutrophil inflammatory network with histological evidence consistent with inflammasome assembly. This signature appears to reflect local thrombus biology rather than clinical disease severity.

## 1. Introduction

Acute ischemic stroke (AIS) caused by large vessel occlusion remains a leading cause of death and long-term disability worldwide despite major advances in reperfusion therapies. Although intravenous thrombolysis and endovascular thrombectomy successfully restore macrovascular patency, many patients continue to exhibit infarct growth and poor functional outcomes despite successful recanalization, highlighting persistent mechanisms of secondary brain injury [1]. Increasing evidence suggests that inflammatory processes contribute to ongoing tissue injury throughout all stages of AIS and critically influence treatment response and clinical outcome [2,3].

Traditionally, cerebral thromboemboli have been regarded primarily as mechanical vascular occlusions. However, recent histopathological analyses have demonstrated that stroke thrombi are highly heterogeneous biological structures composed of distinct erythrocyte-rich and fibrin/platelet-rich regions [4,5,6]. Beyond their structural composition, thrombi contain substantial numbers of leukocytes, inflammatory mediators, and extracellular DNA networks. In particular, neutrophil extracellular traps (NETs) have emerged as important contributors to thrombus organization, thrombolysis resistance, and mechanical thrombectomy failure [7,8,9]. These observations have led to the concept of immunothrombosis, in which inflammatory and coagulation pathways cooperatively promote thrombus formation and stabilization [10].

The NOD-like receptor family pyrin domain-containing protein 3 (NLRP3) inflammasome is a central regulator of sterile inflammation. Following activation, NLRP3 promotes caspase 1-activation, maturation of interleukin-1β and interleukin-18, and cleavage of Gasdermin D (GSDMD), ultimately resulting in pyroptotic cell death and release of danger-associated molecular patterns such as high-mobility group box 1 (HMGB1) [11]. Experimental studies have identified NLRP3 as a major contributor to ischemic brain injury, while pharmacological inhibition of NLRP3 attenuates infarct progression in several preclinical stroke models [12,13,14,15]. Furthermore, recent translational evidence suggests that NLRP3-dependent inflammatory responses are already activated during large vessel occlusion before recanalization [16]. However, whether NLRP3 signaling is present within human cerebral thromboemboli themselves and how it relates to established immunothrombotic mechanisms remain unknown.

Emerging evidence suggests that thrombi are not merely passive vascular occlusions but rather complex immunological structures in which coagulation and inflammation converge [10]. We therefore hypothesized that human stroke thrombi harbor an inflammasome-associated inflammatory network characterized by coordinated activation of neutrophil-driven and pyroptosis-associated pathways. To test this hypothesis, we characterized NLRP3 immunoreactivity in thrombectomy-derived thrombi from patients with acute ischemic stroke and investigated its relationship to thrombus composition, neutrophil accumulation, NETosis, GSDMD and HMGB1 expression. In addition, we examined whether thrombus-associated NLRP3 signatures are linked to the patients’ clinical outcomes.

## 2. Materials and Methods

### 2.1. Patient Cohort and Eligibility Criteria

Thirty-four thrombi retrieved during mechanical thrombectomy from patients with AIS caused by large vessel occlusion were included in this study between 2020 and 2021 as described before [17]. The study was approved by the Ethics Committee of the Medical Faculty of the University of Wuerzburg, Germany (approval nr. 135/17; amended on 16 March 2018, 17 June 2020, and 4 February 2022) and written informed consent was provided by all participants. Eligibility criteria included AIS with a baseline NIHSS score ≥ 6, imaging-confirmed large vessel occlusion involving the distal internal carotid artery (ICA-T), middle cerebral artery M1 segment, or proximal M2 segment, and treatment by mechanical thrombectomy according to current guideline recommendations.

### 2.2. Patient Information

We gathered demographic, clinical, radiographic, and interventional information, encompassing age, gender, symptom onset timing, baseline pharmaceuticals, baseline heart rate and blood pressure, NIHSS at the initial presentation and after 24, 48 and 72 h, the modified Rankin Scale (mRS) baseline, at discharge and at 3 months, ASPECTS at the outset, prior intravenous administration of recombinant tissue plasminogen activator (rt-PA), timing of blood sample collection, and recanalization timing. Recanalization success was determined by the modified treatment in cerebral ischemia (mTICI) scale, considering success for mTICI 2b or mTICI 3.

### 2.3. Immunohistochemistry

Mechanical thrombectomy procedures have been described previously [17]. Retrieved thrombi were embedded in Tissue-Tek OCT compound, cryopreserved, and cut into 10 μm thick sections using a cryostat. Immunofluorescence stainings were performed according to standard protocols [13]. The following primary antibodies were used: mouse anti-human NLRP3/NALP3 mAb (Cryo-2; Adipogen Life Sciences, San Diego, CA, USA; 1:100), rabbit anti-Gasdermin D (E9S1X; Cell Signaling Technology, Danvers, MA, USA; 1:100), mouse anti-human HMGB1 (ab190377, Abcam, Cambridge, UK; 1:50), rabbit anti-human CD66B (ab197678, Abcam, Cambridge, UK; 1:50), rabbit anti-human H3CIT (ab5103, Abcam, Cambridge, UK; 1:50), and mouse anti-human ASC (04-147; Merck; Darmstadt, Germany; 1:100). Nuclei were counterstained with DAPI.

Secondary antibodies included Alexa Fluor 488 goat anti-mouse IgG (1:100, A11001, Invitrogen, Carlsbad, CA, USA), Alexa Fluor 488 donkey anti-rabbit IgG (1:100, A21206, Invitrogen, Carlsbad, CA, USA), Alexa Fluor 488 goat anti-rabbit IgG (1:100, A11008, Invitrogen, Carlsbad, CA, USA), Alexa Fluor 594 goat anti-rabbit IgG (1:100, A21244, Invitrogen, Carlsbad, CA, USA), Alexa Fluor 647 goat anti-mouse IgG (1:100, A21235, Invitrogen, Carlsbad, CA, USA), and Alexa Fluor 647 goat anti-rabbit IgG (1:100, A21245, Invitrogen, Carlsbad, CA, USA). Depending on the staining combination, the appropriate fluorophore-conjugated secondary antibodies were used.

For sequential same-species multiplex staining, unconjugated Fab Fragment Goat Anti-Rabbit IgG (H+L) (111-007-003, Jackson ImmunoResearch, West Grove, PA, USA) and normal rabbit serum (011-000-120, Jackson ImmunoResearch, West Grove, PA, USA) were used.

For qualitative triple immunofluorescence stainings, sequential same-species labeling was performed by first saturating residual antigen-binding sites of the rabbit-specific secondary antibody with normal rabbit serum, followed by masking of the first rabbit primary antibody using unconjugated goat anti-rabbit IgG Fab fragments prior to incubation with the second rabbit primary antibody. For sequential same-species staining, omission of the second rabbit primary antibody after Fab blocking served to verify complete masking of the first rabbit primary antibody.

As negative controls, matched isotype control antibodies and secondary-antibody-only controls were used under the same conditions as the primary antibodies: polyclonal rabbit IgG isotype control antibody (910801, Biolegend, San Diego, CA, USA), monoclonal rabbit IgG isotype control antibody (3900; Cell Signaling Technology, Danvers, MA, USA; 1:100), and mouse IgG2b isotype control (AG-35B-0005-C050, Adipogen Life Sciences, San Diego, CA, USA).

Images were acquired using a Leica DMi8 fluorescence microscope equipped with DMC 2900/DFC 3000G camera control and LAS X software (version 3.1; Leica Microsystems, Wetzlar, Germany). Fluorescence channels were pseudocolored during image processing for optimal visualization. The displayed colors do not necessarily correspond to the original fluorophores used for image acquisition.

Quantitative image analysis was performed using ImageJ software (ImageJ Analysis Software 1.52a, National Institutes of Health, Bethesda, MD, USA). Mean fluorescence intensity was used for NLRP3, HMGB1 and GSDMD quantification because it more accurately reflects heterogeneous expression levels than area-based measurements alone as previously described [13]. NETosis was assessed by quantification of H3CIT-positive areas. CD66B-positive areas, H3CIT-positive areas, fibrin-rich regions, erythrocyte-rich regions and DAPI-positive cellular areas were quantified and expressed as percentage of the total thromboembolus area. To determine the cellular localization of inflammasome-associated signaling, double immunofluorescence stainings for the Apoptosis-associated speck-like protein containing a CARD (ASC) and CD66B were performed. For each thrombus, the proportion of CD66B-positive neutrophils exhibiting ASC immunoreactivity was determined by manual cell counting.

Fibrin-rich and hemoglobin-rich thrombus regions were identified based on hematoxylin and eosin (H&E) staining. For each thrombus, three consecutive sections encompassing the entire thrombus area were analyzed, and the measurements were averaged. Quantification was performed independently by two blinded investigators. All quantitative analyses were performed exclusively on double immunofluorescence stainings. Triple immunofluorescence stainings were generated solely for qualitative visualization of marker co-localization in the representative figures and were not used for quantitative image analysis.

For fluorescence quantification, the entire thrombus area on each section was manually delineated as a region of interest (ROI) using ImageJ software. Empty spaces, tissue artifacts, sectioning defects and areas lacking thrombus material were excluded from the analysis. Background fluorescence was determined in cell-free regions without specific staining and subtracted from all measurements to minimize nonspecific signal contribution. Mean fluorescence intensity was subsequently quantified within the delineated thrombus ROI after background subtraction. All images were acquired using identical microscope settings and analyzed using predefined thresholds to ensure comparability between samples.

### 2.4. Statistical Analysis

Statistical analyses were performed using GraphPad Prism 10 (GraphPad Software, San Diego, CA, USA). Data were tested for Gaussian distribution using the D’Agostino–Pearson omnibus normality test. Associations between continuous variables were assessed using Pearson correlation analyses. Multiple linear regression analyses were performed to determine whether associations between NLRP3 expression and inflammatory thrombus markers remained significant after adjustment for fibrin content. Correlation matrices and network visualizations were generated based on significant pairwise correlations. Two-sided *p* values < 0.05 were considered statistically significant.

## 3. Results

### 3.1. Patient Cohort and Thrombus Collection

We included thirty-four thrombi retrieved by mechanical thrombectomy from patients with AIS in the study. Histological and immunofluorescence analyses were performed to characterize thrombus composition and inflammatory marker expression. Clinical and demographic characteristics of the study cohort are summarized in Table 1.

### 3.2. NLRP3 Is Present in Human Ischemic Stroke Thrombi and Exhibits Marked Spatial Heterogeneity

**Figure 1:**
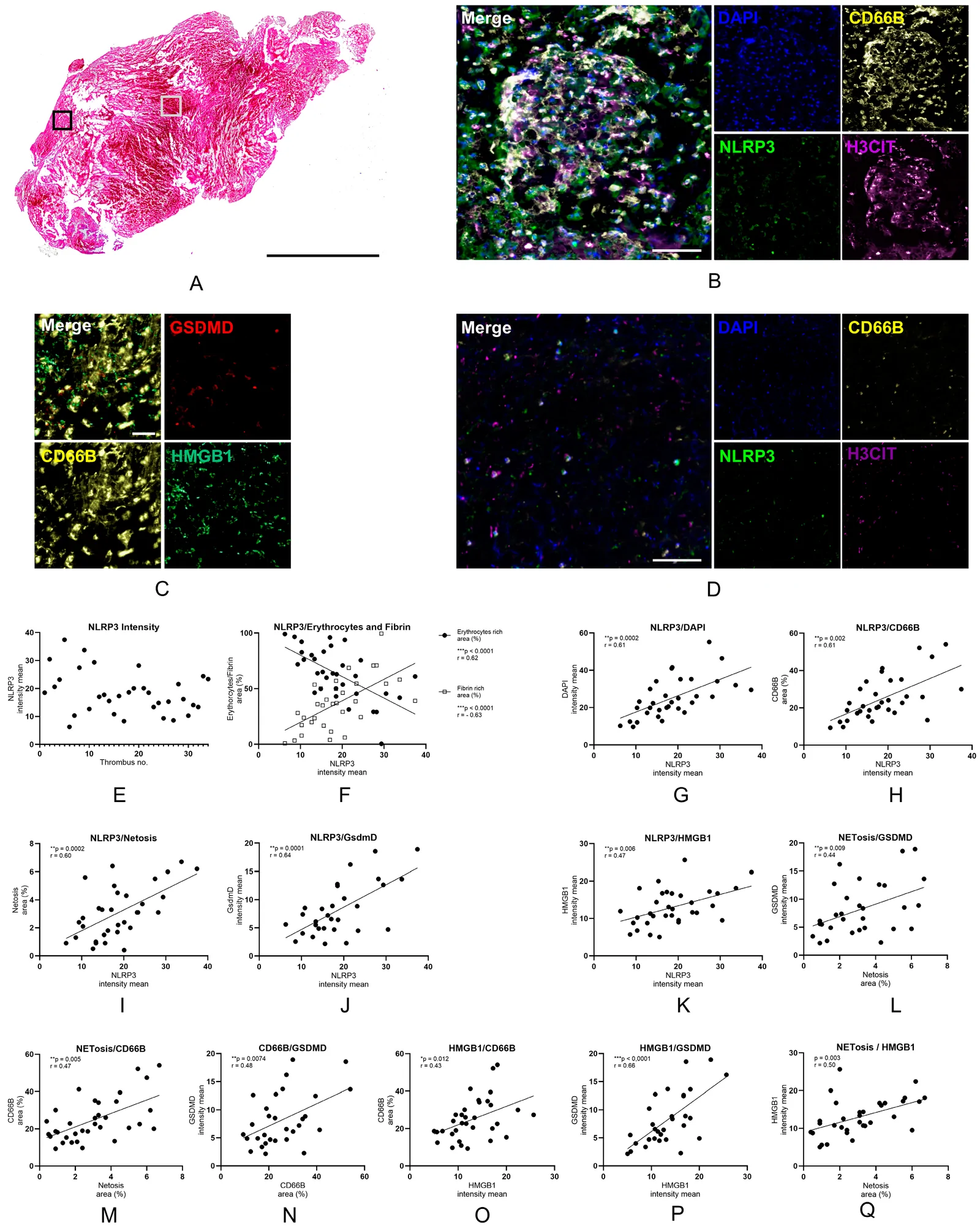
NLRP3 immunoreactivity is associated with inflammatory thrombus signatures in human ischemic stroke thrombi. Representative H&E staining of a thrombectomy-derived ischemic stroke thrombus illustrating the marked spatial heterogeneity of thrombus composition and indicating regions selected for immunofluorescence analyses (**A**). Representative immunofluorescence images obtained from the fibrin- and leukocyte-rich region highlighted in black in (**A**) demonstrate strong expression of NLRP3, CD66B, and H3CIT (**B**), as well as HMGB1 and GSDMD (**C**). In contrast, the erythrocyte-rich region highlighted in gray in (**A**) exhibited only low inflammatory marker expression (**D**). NLRP3 is shown in green, CD66B in yellow, H3CIT in magenta, and DAPI in blue. HMGB1 is shown in aquamarine and GSDMD in red. Quantification of NLRP3 immunoreactivity across all analyzed thrombi is shown in (**E**). Correlations between NLRP3 immunoreactivity and thrombus composition (**F**), cellularity assessed by DAPI staining (**G**), neutrophil abundance assessed by CD66B staining (**H**), NETosis assessed by H3CIT staining (**I**), GSDMD expression (**J**), and HMGB1 expression (**K**) are displayed. Additional correlations among CD66B, NETosis, HMGB1, and GSDMD are presented in panels (**L**–**Q**). Pearson correlation coefficients (r) and corresponding *p* values are indicated in the respective panels. Scale bars = 500 μm in (**A**), 50 μm in (**B**,**D**) and 30 µm in (**C**). Significance is shown as follows: * = *p* < 0.05, ** = *p* < 0.01, *** = *p* < 0.001.

NLRP3 immunofluorescence was detected in all analyzed thrombi and showed substantial inter- and intrathrombus heterogeneity. Representative images revealed focal accumulation of NLRP3-positive signal within distinct thrombus regions, whereas other areas showed little or no staining. NLRP3 preferentially accumulated in fibrin-rich, highly cellular regions enriched for inflammatory cells (Figure 1A,B,D). Quantification of NLRP3 immunofluorescence intensity demonstrated considerable variability across individual thrombi (Figure 1E). NLRP3 immunofluorescence intensity correlated positively with fibrin-rich areas (r = 0.62, *p* < 0.0001) and negatively with erythrocyte-rich regions (r = −0.63, *p* < 0.0001) (Figure 1F). Furthermore, NLRP3 immunofluorescence signal correlated with thrombus cellularity assessed by DAPI staining (r = 0.61, *p* = 0.0002) and with neutrophil abundance measured by CD66B staining (r = 0.61, *p* = 0.002) (Figure 1G,H).

### 3.3. NLRP3 Is Embedded Within a Coordinated Inflammatory Thrombus Network

**Figure 2:**
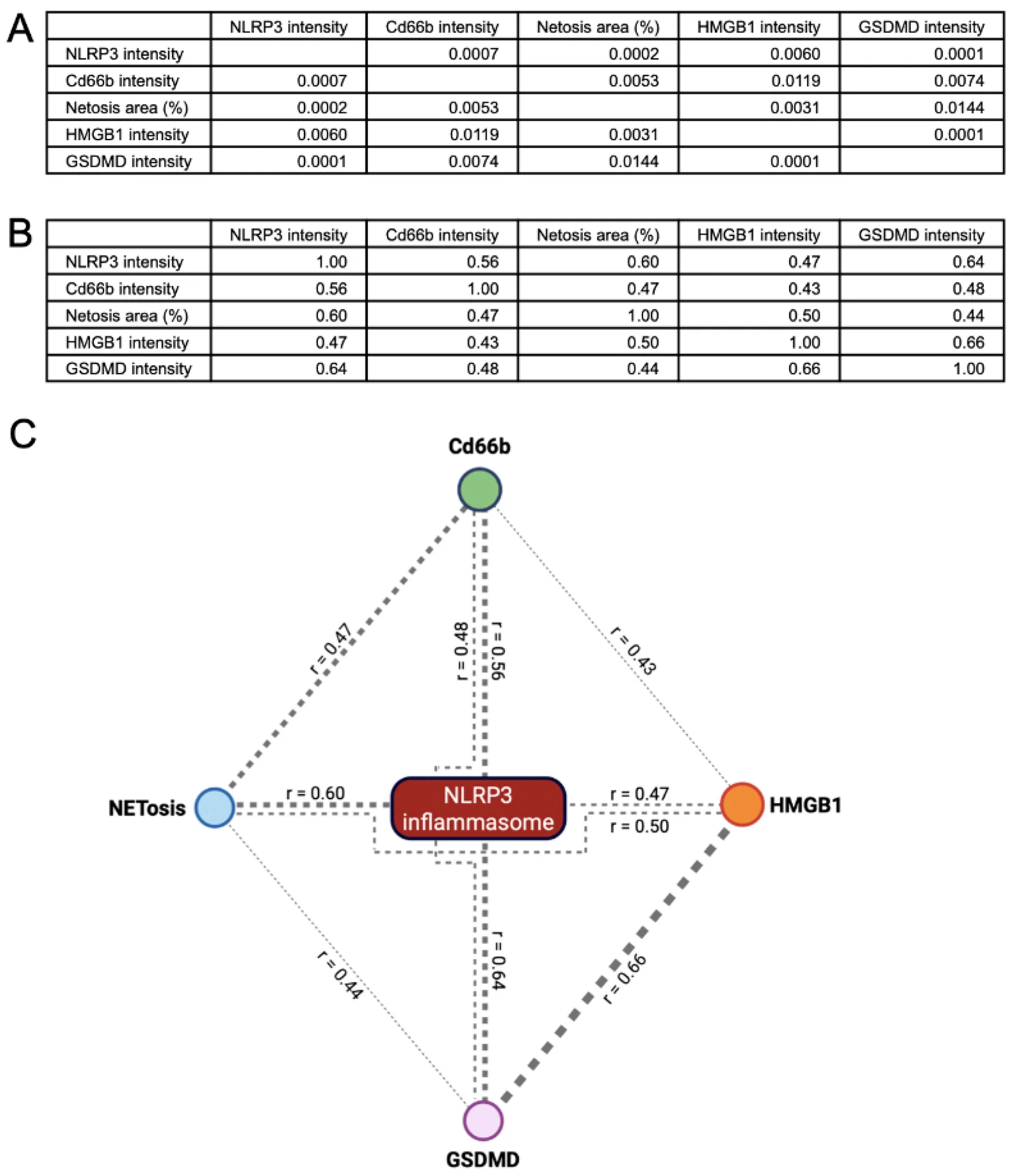
Correlation matrix and network analysis of inflammatory thrombus markers. (**A**) Pairwise *p* values and (**B**) Pearson correlation coefficients (r) for associations between NLRP3 immunoreactivity, CD66B, NETosis (H3CIT), HMGB1, and GSDMD. (**C**) Network visualization summarizing significant correlations within the thrombus inflammatory marker panel. Edge labels indicate Pearson correlation coefficients (r), and line thickness reflects the strength of the respective association. Only statistically significant correlations are displayed.

NLRP3 immunoreactivity correlated with NETosis (H3CIT; r = 0.60, *p* = 0.0002), HMGB1 expression (r = 0.47, *p* = 0.006), and GSDMD expression (r = 0.64, *p* = 0.0001). CD66B abundance correlated with NETosis (r = 0.47, *p* = 0.005), HMGB1 (r = 0.43, *p* = 0.01), and GSDMD (r = 0.48, *p* = 0.0074). HMGB1 correlated with NETosis (r = 0.50, *p* = 0.003) and GSDMD (r = 0.66, *p* = 0.0001), while NETosis correlated with GSDMD (r = 0.44, *p* = 0.01) (Figure 1C,I–P). Correlation matrix and network analyses summarized the relationships among NLRP3, CD66B, HMGB1, H3CIT, and GSDMD (Figure 2).

### 3.4. ASC Expression in Thrombus-Associated Neutrophils

**Figure 3:**
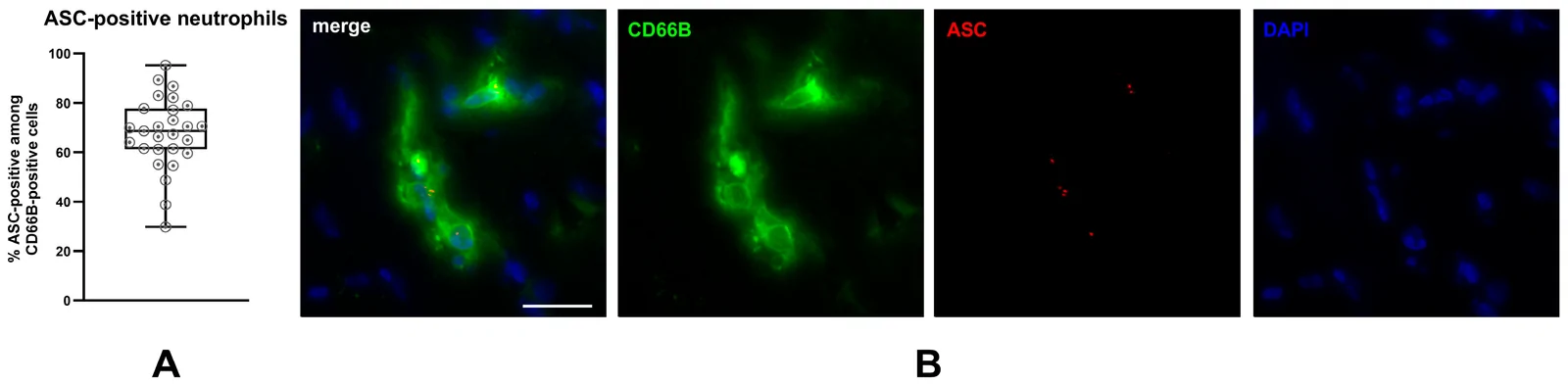
ASC expression in thrombus-associated neutrophils. (**A**) Quantification of the proportion of CD66B-positive neutrophils expressing ASC immunoreactivity across all analyzed thrombi (mean 67.7%). Each dot represents one thrombus. (**B**) Representative immunofluorescence images demonstrate ASC expression (red) in CD66B-positive neutrophils (green) within human ischemic stroke thrombi. DAPI in blue. Scale bar = 20 µm.

To further substantiate inflammasome-associated signaling in thrombus-associated neutrophils, ASC immunofluorescence staining was performed. Quantitative analysis demonstrated that a mean of 67.7% of CD66B-positive neutrophils exhibited ASC immunoreactivity across all analyzed thrombi (Figure 3A). Immunofluorescence demonstrated ASC-positive specks within CD66B-positive neutrophils (Figure 3B). Notably, the proportion of ASC-positive neutrophils did not correlate with overall CD66B immunoreactivity (r = 0.188, *p* = 0.287), fibrin area (r = 0.005, *p* = 0.977), or erythrocyte-rich area (r = − 0.005, *p* = 0.977), indicating that inflammasome-associated ASC positivity is a consistent feature of thrombus-associated neutrophils across thrombi with different structural compositions.

### 3.5. Associations Between NLRP3 and Inflammatory Signaling Persist Independently of Fibrin Content

Multivariable linear regression analyses including NLRP3 immunoreactivity and fibrin content demonstrated independent associations of NLRP3 with NETosis, HMGB1, GSDMD, and CD66B. Fibrin content was not independently associated with NETosis, HMGB1, or CD66B but showed a significant association with GSDMD signal intensity (Supplementary Table S1).

### 3.6. Thrombus-Associated NLRP3 Expression Is Not Associated with Clinical Parameters

**Figure 4:**
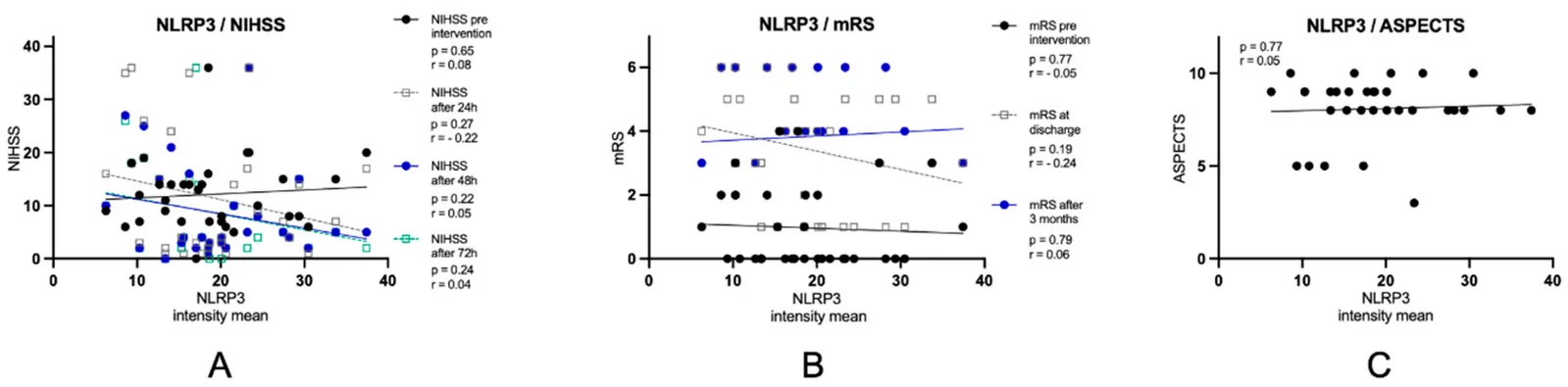
Thrombus-associated NLRP3 immunoreactivity is not associated with outcome measures. Associations between thrombus NLRP3 immunoreactivity with baseline stroke severity assessed by the NIHSS (**A**), functional outcome measured by the modified Rankin Scale (mRS) (**B**), and infarct extent estimated by ASPECTS (**C**) are shown. Pearson correlation coefficients (r) and corresponding *p* values are indicated in the respective panels.

Thrombus NLRP3 immunoreactivity was not associated with baseline stroke severity (NIHSS), infarct extent (ASPECTS), or functional outcome assessed by mRS at follow-up (Figure 4).

## 4. Discussion

The present study identifies an NLRP3-centered inflammatory network within human ischemic stroke thrombi. NLRP3 immunoreactivity was enriched in fibrin-rich and highly cellular regions and was closely associated with neutrophil abundance, NETosis, HMGB1, and GSDMD. In addition, ASC-positive specks were frequently detected in thrombus-associated neutrophils.

After adjustment for fibrin content, NLRP3 remained associated with CD66B, NETosis, HMGB1, and GSDMD, indicating that the inflammatory signature cannot be explained solely by preferential localization within fibrin-rich thrombus regions.

The close relationship between NLRP3, NETosis, HMGB1, and GSDMD is consistent with established links between inflammasome assembly, neutrophil-mediated inflammation, DAMP release, and pyroptosis-associated signaling. The frequent detection of ASC-positive specks in CD66B-positive neutrophils provides additional histological evidence consistent with inflammasome assembly within the neutrophil compartment. Notably, the proportion of ASC-positive neutrophils was unrelated to overall neutrophil abundance or thrombus composition, suggesting that this feature is not confined to a specific structural thrombus phenotype. However, the observational design precludes conclusions regarding causality or pathway directionality. These observations are consistent with previous studies demonstrating inflammasome-associated proteins and NET-rich inflammatory microdomains within thrombectomy-derived stroke thrombi [6,7,8]. Inflammasome proteins have previously been identified in NET-containing thrombus regions, supporting a close spatial relationship between inflammasome signaling and neutrophil-driven thrombus inflammation [18]. Proteomic analyses further suggest that NET-rich thrombi represent a distinct inflammatory thrombus phenotype characterized by coordinated activation of multiple innate immune pathways [19].

Despite its close association with neutrophils, NETosis, HMGB1, and GSDMD, thrombus NLRP3 was not associated with stroke severity, imaging findings, or functional outcome. This stands in sharp contrast to our previous study of post-occlusive pial blood, where NLRP3-related inflammatory activity was linked to infarct progression and clinical outcome [16]. These observations suggest that thrombus-associated NLRP3 primarily reflects local thrombus biology rather than mechanisms directly driving tissue injury and recovery.

Several limitations should be acknowledged. First, marker expression was assessed by immunofluorescence, providing valuable spatial information but not a direct functional readout of inflammasome activity. Although cleavage-specific antibodies against activated caspase-1 and the N-terminal fragment of Gasdermin D are conceptually attractive, they did not yield sufficiently robust and scientifically interpretable quantitative data in archived human thrombus tissue. Therefore, ASC was selected as the most reliable marker of inflammasome assembly. Second, the present study deliberately focused on the neutrophil compartment because our primary hypothesis addressed a neutrophil–NETosis–pyroptosis-associated inflammatory network. This rationale was supported by the established role of neutrophils in immunothrombosis and by our previous demonstration of neutrophil-associated NLRP3 activation during hyperacute ischemic stroke [16]. Consequently, monocytes and monocyte/macrophage-lineage cells were not systematically investigated and should be addressed in future studies to define their relative contribution to thrombus-associated inflammasome signaling. Finally, the sample size limits more extensive adjustment for clinical and procedural variables.

## 5. Conclusions

Human ischemic stroke thrombi harbor an NLRP3-associated inflammatory network linked to neutrophils, NETosis, HMGB1, and GSDMD. The additional detection of ASC-positive specks provides complementary histological evidence consistent with inflammasome assembly within thrombus-associated neutrophils, supporting the concept of local immunothrombotic inflammatory signaling rather than a marker of clinical stroke severity.

## Figures and Tables

**Table 1:** Demographic, imaging, treatment, and outcome-related patient characteristics. Values are presented as number (percentage) for categorical variables and median (IQR) for continuous variables. ASPECTS = Alberta Stroke Program Early CT Score; IQR = interquartile range; i.v. rt-PA = intravenous recombinant tissue plasminogen activator; NIHSS = National Institutes of Health Stroke Scale; mRS = modified Rankin Scale.

Demographics	
Age, yr, median (IQR)	79 (70–86) (N = 34)
Female sex, n (%)	20 (59%) (N = 34)
Antiplatelet medication at onset, n (%)	10 (29%) (N = 34)
Anticoagulation at onset, n (%)	10 (29%) (N = 34)
Patients with previous stroke, n (%)	8 (24%) (N = 34)
**Imaging Data**	
Baseline ASPECTS, median (IQR)	8 (8–9) (N = 34)
**Treatment**	
i.v. rt-PA, n (%)	18 (53%) (N = 34)
Intervention: Onset-to-recanalization, min, median (IQR)	362 (233–496) (N = 34)
**Outcome**	
NIHSS pre intervention, median (IQR)	11 (7–15) (N = 34)
NIHSS after 24 h, median (IQR)	7 (2–17) (N = 32)
NIHSS after 48 h, median (IQR)	7 (2–17) (N = 32)
NIHSS after 72 h, median (IQR)	5 (2–15) (N = 32)
mRS at discharge, median (IQR)	4 (1–5) (N = 33)
mRS after 3 months, median (IQR)	4 (3–6) (N = 25)

## Data Availability

The raw data supporting the conclusions of this article will be made available by the corresponding author on request.

## Supplementary Materials

The following supporting information can be downloaded at https://www.mdpi.com/article/10.3390/cells15171597/s1. Table S1: Multivariable linear regression analyses.

## Abbreviations

The following abbreviations are used in this manuscript:

AIS	Acute ischemic stroke
ASC	Apoptosis-associated speck-like protein containing a CARD
ASPECTS	Alberta Stroke Program Early CT Score
CD66B	Cluster of Differentiation 66B
DAMP	Danger-associated molecular pattern
GSDMD	Gasdermin D
H&E	Hematoxylin and eosin
HMGB1	High-mobility group box 1
H3CIT	Citrullinated histone H3
ICA-T	Internal carotid artery terminus
mRS	Modified Rankin Scale
mTICI	Modified Thrombolysis in Cerebral Infarction
MT	Mechanical thrombectomy
NET	Neutrophil extracellular trap
NETosis	Neutrophil extracellular trap formation
NIHSS	National Institutes of Health Stroke Scale
NLRP3	NOD-like receptor family pyrin domain-containing protein 3
ROI	Region of interest
