# Supplementary material for: NLRP3 Marks an Inflammatory Neutrophil–NETosis–Pyroptosis Network in Human Ischemic Stroke Thrombi
Source: Cells. 2026 Sep 2;15(17):1597. doi: 10.3390/cells15171597 (PMC13565499; doi:10.3390/cells15171597)
Supplement: Supplementary file 1 [file cells-15-01597-s001.zip › cells-4465689-supplementary.pdf]

Supplement

Supplementary Table S1: Multivariable linear regression analyses

Dependent variable	Predictor	β coefficient	95% CI	p value	Model R^2	Overall Model p
NETosis	NLRP3	0.176	0.084–0.269	0.0005	0.382	0.0007
	Fibrin	-0.015	-0.045–0.016	0.33		
HMGB1	NLRP3	0.431	0.186–0.677	0.0011	0.480	0.0001
	Fibrin	-0.019	-0.074–0.036	0.47		
Gasdermin D	NLRP3	0.110	0.007–0.213	0.0386	0.496	0.0001
	Fibrin	0.025	0.001–0.048	0.0434		
Cd66b	NLRP3	0.999	0.404-1.594	0.0018	0.369	0.001
	Fibrin	-0.025	-0.218-0.168	0.7591		

Supplementary Table S1. Multivariable linear regression analyses examining the independent associations of NLRP3 immunoreactivity and fibrin content with inflammatory thrombus markers. Regression coefficients (β), 95% confidence intervals (CI), p values, model R^2 values, and overall model p values are reported for each model. NETosis, HMGB1, Gasdermin D, and CD66b served as dependent variables; NLRP3 immunoreactivity and fibrin content were included as independent variables.
